# Abdominal pain in patients with inflammatory bowel disease: association with single-nucleotide polymorphisms prevalent in irritable bowel syndrome and clinical management

**DOI:** 10.1186/s12876-021-01622-x

**Published:** 2021-02-05

**Authors:** Martina Ledergerber, Brian M. Lang, Henriette Heinrich, Luc Biedermann, Stefan Begré, Jonas Zeitz, Niklas Krupka, Andreas Rickenbacher, Matthias Turina, Thomas Greuter, Philipp Schreiner, René Roth, Alexander Siebenhüner, Stephan R. Vavricka, Gerhard Rogler, Niko Beerenwinkel, Benjamin Misselwitz, the Swiss IBD Cohort Study Group, Claudia Anderegg, Peter Bauerfeind, Christoph Beglinger, Stefan Begré, Dominique Belli, José M. Bengoa, Luc Biedermann, Beat Bigler, Janek Binek, Mirjam Blattmann, Stephan Boehm, Jan Borovicka, Christian P. Braegger, Nora Brunner, Patrick Bühr, Bernard Burnand, Emanuel Burri, Sophie Buyse, Matthias Cremer, Dominique H. Criblez, Philippe de Saussure, Lukas Degen, Joakim Delarive, Christopher Doerig, Barbara Dora, Gian Dorta, Mara Egger, Tobias Ehmann, Ali El-Wafa, Matthias Engelmann, Jessica Ezri, Christian Felley, Markus Fliegner, Nicolas Fournier, Montserrat Fraga, Pascal Frei, Remus Frei, Michael Fried, Florian Froehlich, Christian Funk, Raoul Ivano Furlano, Suzanne Gallot-Lavallée, Martin Geyer, Marc Girardin, Delphine Golay, Tanja Grandinetti, Beat Gysi, Horst Haack, Johannes Haarer, Beat Helbling, Peter Hengstler, Denise Herzog, Cyrill Hess, Klaas Heyland, Thomas Hinterleitner, Philippe Hiroz, Claudia Hirschi, Petr Hruz, Rika Iwata, Res Jost, Pascal Juillerat, Vera Kessler Brondolo, Christina Knellwolf, Christoph Knoblauch, Henrik Köhler, Rebekka Koller, Claudia Krieger-Grübel, Gerd Kullak-Ublick, Patrizia Künzler, Markus Landolt, Rupprecht Lange, Frank Serge Lehmann, Andrew Macpherson, Philippe Maerten, Michel H. Maillard, Christine Manser, Michael Manz, Urs Marbet, George Marx, Christoph Matter, Valérie McLin, Rémy Meier, Martina Mendanova, Christa Meyenberger, Pierre Michetti, Benjamin Misselwitz, Darius Moradpour, Bernhard Morell, Patrick Mosler, Christian Mottet, Christoph Müller, Pascal Müller, Beat Müllhaupt, Claudia Münger-Beyeler, Leilla Musso, Andreas Nagy, Michaela Neagu, Cristina Nichita, Jan Niess, Natacha Noël, Andreas Nydegger, Nicole Obialo, Carl Oneta, Cassandra Oropesa, Ueli Peter, Daniel Peternac, Laetitia Marie Petit, Franziska Piccoli-Gfeller, Julia Beatrice Pilz, Valérie Pittet, Nadia Raschle, Ronald Rentsch, Sophie Restellini, Jean-Pierre Richterich, Sylvia Rihs, Marc Alain Ritz, Jocelyn Roduit, Daniela Rogler, Gerhard Rogler, Jean-Benoît Rossel, Markus Sagmeister, Gaby Saner, Bernhard Sauter, Mikael Sawatzki, Michela Schäppi, Michael Scharl, Martin Schelling, Susanne Schibli, Hugo Schlauri, Sybille Schmid Uebelhart, Jean-François Schnegg, Alain Schoepfer, Frank Seibold, Mariam Seirafi, Gian-Marco Semadeni, David Semela, Arne Senning, Marc Sidler, Christiane Sokollik, Johannes Spalinger, Holger Spangenberger, Philippe Stadler, Michael Steuerwald, Alex Straumann, Bigna Straumann-Funk, Michael Sulz, Joël Thorens, Sarah Tiedemann, Radu Tutuian, Stephan Vavricka, Francesco Viani, Jürg Vögtlin, Roland Von Känel, Alain Vonlaufen, Dominique Vouillamoz, Rachel Vulliamy, Jürg Wermuth, Helene Werner, Paul Wiesel, Reiner Wiest, Tina Wylie, Jonas Zeitz, Dorothee Zimmermann

**Affiliations:** 1 Department of Gastroenterology, University Hospital Zurich (USZ), Zurich University, Zurich, Switzerland; 2 Department of Biosystems Science and Engineering, ETH Basel, Basel, Switzerland; 3 SIB Swiss Institute of Bioinformatics, Basel, Switzerland; 4 Department of Biomedical Research, Neurology, Inselspital and University Clinic of Bern, Bern, Switzerland; 5 Center of Gastroenterology, Clinic Hirslanden, Zurich, Switzerland; 6 Department of Visceral Surgery and Medicine, Inselspital Bern, University of Bern, Bern, Switzerland; 7 Department of Visceral Surgery, University Hospital Zurich (USZ), Zurich, Switzerland; 8 Department of Oncology, Center of Hematology and Oncology University Hospital Zurich (USZ), Zurich University, Zurich, Switzerland

**Keywords:** Single-nucleotide polymorphisms, Abdominal pain, Inflammatory bowel disease, Ulcerative colitis, Crohn’s disease, Irritable bowel syndrome

## Abstract

**Background:**

Abdominal pain is a frequent symptom in patients with inflammatory bowel disease (IBD) including Crohn’s disease (CD) and ulcerative colitis (UC). Pain can result from ongoing inflammation or functional disorders imitating irritable bowel syndrome (IBS). Several single-nucleotide polymorphisms (SNPs) have been associated with IBS. However, the impact of IBS genetics on the clinical course of IBD, especially pain levels of patients remains unclear.

**Methods:**

Data of 857 UC and 1206 CD patients from the Swiss IBD Cohort Study were analysed. We tested the association of the maximum of the abdominal pain item of disease activity indices in UC and CD over the study period with 16 IBS-associated SNPs, using multivariate ANOVA models.

**Results:**

In UC patients, the SNPs rs1042713 (located on the ADRB2 gene) and rs4663866 (close to the HES6 gene) were associated with higher abdominal pain levels (*P* = 0.044; *P* = 0.037, respectively). Abdominal pain was not associated with any markers of patient management in a model adjusted for confounders. In CD patients, higher levels of abdominal pain correlated with the number of physician contacts (*P* < 10^–15^), examinations (*P* < 10^–12^), medical therapies (*P* = 0.023) and weeks of hospitalisation (*P* = 0.0013) in a multivariate model.

**Conclusions:**

We detected an association between maximal abdominal pain in UC patients and two IBS-associated SNPs. Abdominal pain levels had a pronounced impact on diagnostic and therapeutic procedures in CD but not in UC patients.

## Background

Inflammatory bowel disease (IBD) comprise inflammatory conditions of the intestinal tract including Crohn’s disease (CD) and ulcerative colitis (UC). The clinical course of IBD is variable, ranging from very mild to severe and life-threatening disease or complications [1]. Identification of reliable predictors for the clinical course of IBD would strongly improve patient management.

Genetic predisposition plays a prominent role in IBD pathogenesis. Genome wide association studies (GWAS) identified more than 240 single-nucleotide polymorphisms (SNPs) associated with the risk for IBD [2–4]. However, in an extensive analysis, even the combined genetic information was largely unable to predict the clinical course of IBD [5]. Therefore, it remains unclear which genetic markers determine patient symptoms, need for therapy and outcome.

Pain is a frequent problem in IBD, affecting approximately 70% of patients and it remains a long-standing problem for every second patient [6]. It is most frequently located in the abdomen and associated with a reduced quality of life (QoL) [7]. The severity of abdominal pain is an integral marker of many scales rating IBD disease activity, including the modified Truelove and Witts disease activity index (MTWAI) for UC [8] and the Crohn’s disease activity index (CDAI) [9] or PRO-2 [10] for CD. Abdominal pain is most severe when acute intestinal inflammation is present but many IBD patients with endoscopic remission continue to experience abdominal pain [6, 11].

Abdominal pain in the absence of active inflammation is also characteristic for irritable bowel syndrome (IBS). IBS is a functional gastrointestinal disorder (FGID) and defined by the Rome IV criteria as recurrent abdominal pain at least once a week over the last three months and two or more of the following criteria: relation to defecation, association with changes in stool frequency or changes in stool form [12]. In a meta-analysis, IBS-like symptoms were reported by almost 40% of IBD patients in remission [13], compared to 20% in the general population [14, 15].

Several SNPs have been analysed in the context of IBS in large GWAS and meta-analyses [16–20]. The role of these “IBS-SNPs” in IBS pathogenesis has not yet been fully understood. Since abdominal pain is the leading symptom in IBS, genetic mechanisms contributing to abdominal pain in IBS might also be relevant for levels of abdominal pain in IBD patients. A potential role of IBS-SNPs on the level of pain in patients with IBD has not yet been investigated.

Even though IBD and IBS are fundamentally different conditions, some pathways of pathogenesis including alterations in gut permeability, microbiota, inflammation, enteric nervous system, gut brain axis and psychological factors are partially shared [21]. Moreover, stimulators for abdominal pain such as tension and strong contractions in the gut, cytokines and an increased sensitivity towards visceral stimuli are likely relevant in both conditions [21–26]. It is therefore reasonable to assume, that IBS-associated genetic polymorphisms will also be relevant in IBD. Since pain remains the clinical hallmark of IBS, we hypothesize that IBS-associated polymorphisms will impact on the level of pain in IBD.

In the Swiss IBD cohort study (SIBDC) extensive clinical information and genetic information are available. The primary aim of this study is to test for a possible association of IBS-SNPs and the level of abdominal pain in patients with IBD. The second goal is to explore the effects of abdominal pain on the disease management.

## Methods

### Patients

The SIBDC has been collecting longitudinal data of IBD patients from all parts of Switzerland since 2006. Patients are followed up yearly, using both a physician and a patient questionnaire. The design and goals of the SIBDC have been described elsewhere [27].

### DNA genotyping and selection of SNPs

In 2015, in a comprehensive effort, the genotype of 379 SNPs was determined in all SIBDC patients consenting to genetic analysis provided sufficient biomaterial was available. Selected SNPs comprise genetic markers associated with IBD risk, smoking, primary sclerosing cholangitis, anxiety, depression and pain after a thorough literature search. Genotyping of the SIBDC-SNPs was performed by Matrix-Assisted Laser Desorption/Ionisation–Time of Flight Mass Spectrometry (MALDI-TOF–MS) based SNP genotyping [28]. The complete list of SNPs available in the SIBDC data base is provided as Additional file 2. Serotonin transporter gene (SERT) polymorphisms (S/S, L/S and L/L) associated with IBS [29, 30] could not be determined by MALDI-TOF–MS for technical reasons.

Our selection of SNPs was based on SNPs previously identified in three GWAS [16–18] and two systematic reviews [19, 20]. To account for the more stringent SNP selection in GWAS, we performed a sensitivity analysis which excluded all SNPs not identified by GWAS. 16 SNPs identified in the above-mentioned five studies were also present in the SIBDC database and were selected for further analysis. No linkage disequilibrium (LD) was detected between the 16 selected SNPs (not shown).

### IBD disease characteristics, socio-demographic and psychological measures

Clinical, epidemiological, socio-demographic and psychosocial data were extracted in autumn 2015 from the SIBDC database. Patient characteristics include gender, age at diagnosis, disease duration, time interval between IBD diagnosis and SIBDC enrolment, ethnicity, smoking habits [31], education, marital status, physical activity, alcohol consumption, initial disease location and extent of disease, family history of IBD and extraintestinal manifestations (EIM). To assess mental health the hospital anxiety and depression scale (HADS) was used.

The primary endpoint of our study was *maximal abdominal pain*, assessed with the abdominal pain item from the CDAI for CD patients and the MTWAI for UC patients, respectively. Both scores distinguish four levels of abdominal pain intensity: none, mild, moderate and severe. We selected the highest reported pain score (i.e. maximum abdominal pain) over the entire observation period. We favoured *maximal abdominal pain* since it is probably the measure closest associated with the main interest of our study: pain typical for IBD during a flare. During a flare, contacts to the health system is frequent and the likelihood of an assessment for SIBDC is high. During these episodes, the highest pain levels are expected. Using maximal abdominal therefore maximises the chances to focus the assessment on pain due to acute inflammation. Moreover, maximal abdominal pain has been identified in a previous study as having the strongest impact on quality of life [32].

We also used median abdominal pain (i.e. median of all reported abdominal pain values over time) as a secondary readout in a sensitivity analysis. This measure likely integrates assessments during a flare as well as during remission. During remission, abdominal pain might be absent (in the absence of an inflammatory stimulus) or due to post-inflammatory IBS, which is not the focus of our study. In further sensitivity analyses, we also tested pain ever (moderate or severe vs. none or mild abdominal pain) as outcomes. To assess *disease activity*, we calculated CDAI for CD and MTWAI for UC, according to SIBDC follow-up questionnaires.

For the *number of physician contacts* we calculated all visits to the primary care physician, gastroenterologist, hospital ambulatories and other doctors, starting three months before enrolment until data extraction.

The *overall number of medical examinations* (abdominal ultrasound, X-rays, CT-scan, MRI, colonoscopies) was used to characterize the vigour of diagnostic efforts. In an additional analysis, the overall number of medical examinations was adjusted for the number of visits.

As a simple summary measure for medical treatments, we added the *number of medical therapies* ever used during the observation period in each patient. The following six classes of drugs were considered: (1) oral/local prednisone or budesonide, (2) purine analogues (azathioprine, mercaptopurine), (3) sulfasalazine and oral/local 5-amino salicylic acid, (4) methotrexate, (5) TNF-inhibitors (infliximab, adalimumab, certolizumab) and (6) calcineurin inhibitors (cyclosporine, tacrolimus). No SIBDC patient had received vedolizumab or golimumab at time of data extraction. Additionally, the *use of TNF-inhibitors* (yes or no) was analysed separately.

To calculate the *duration of hospitalisation*, all weeks spent hospitalised, from 12 months prior to enrolment until data extraction, were summarized. Endpoints for the *quality of life* were the inflammatory disease questionnaire (IBDQ) [33, 34] and the Short Form 36 Health Survey (SF-36) [35, 36].

### Statistical analysis

Data were imported into the statistical program R version 3.3.3 [37]. To assess the relationship between IBS-associated SNPs and the endpoint of interest we first screened for an association of genotype and endpoint using univariate regression. For the multivariate analysis, we performed step-wise model selection, considering disease characteristics, demographic features and psychosocial factors as predictors. Parameters were systematically eliminated by comparing models using the Akaike information criterion (AIC). AIC is a measure for the goodness-of-fit of a given model that takes model complexity into account with a preference for models with a lower number of parameters. We then included SNPs and covariates in a multivariate analysis of variance (ANOVA) model for prediction of our endpoints of interest. We performed model-based t-tests (contrasts) to assess individual associations between SNPs and endpoints while controlling for covariates. We used Bonferroni and Benjamini–Hochberg correction [38] to correct for multiple testing. A corrected p-value (q) < 0.1 was considered significant.

## Results

### Study population and genetic information

For our analysis, clinical, epidemiological and genetic data of 2063 individuals with IBD were available. 1206 of those patients were diagnosed with CD and 857 with UC. Table 1 provides an overview of the study population; our study population comprises patients with mild, moderate and severe disease. The collected data of up to nine years of follow-up was analysed.

**Table 1:** Basic data and disease characteristics

	Overall	CD	UC
	n = 2063	n = 1206	n = 857
*Basic epidemiological and sociodemographic characteristics*			
Sex: Female; n (%)	1007 (48.8)	621 (51.5)	386 (45.0)
Age at diagnosis, median (sd)	31.2 (13.6)	29.9 (13.5)	33.0 (13.5)
Age at enrolment (years)	41.0 (14.7)	40.5 (15)	41.8 (14.3)
Disease duration (years)	14.6 (9.9)	15.4 (10.5)	13.4 (9.1)
Duration between diagnosis and enrolment (years)	9.9 (9.5)	10.6 (10)	8.76 (8.70)
Caucasian; n (%)	0.8 (0.4)	0.8 (0.4)	0.8 (0.4)
Higher education; n (%)	447 (27.1)	237 (25.2)	210 (29.7)
Swiss citizenship; n (%)	1271 (79.9)	732 (80.1)	539 (79.7)
Married; n (%)	779 (48.1)	404 (43.8)	375 (54.0)
Low physical activity (%)	652 (40.9)	406 (44.8)	246 (35.9)
Ever smoke; n (%)	955 (46.3)	698 (57.9)	257 (30.0)
Alcohol: rarely or never; n (%)	938 (57.0)	559 (59.4)	379 (53.7)
Alcohol: frequent (once a day); n (%)	111 (6.7)	73 (7.8)	38 (5.4)
Family history of CD; n (%)	172 (8.9)	143 (12.7)	29 (3.6)
Family history of UC; n (%)	110 (5.7)	37 (3.3)	73 (9.2)
*Disease characteristics*			
Disease localisation CD			
Upper gastrointestinal tract		11 (0.9)	
Ileal		266 (22.1)	
Ileocolonic		572 (47.4)	
Colonic		263 (21.8)	
Disease localisation UC			
Proctitis			134 (15.6)
Left-sided colitis			286 (33.3)
Pancolitis			355 (41.3)
Extraintestinal manifestations, any; n (%)	1181 (57.2)	738 (61.2)	443 (51.6)
Extraintestinal manifestation: arthritis/arthralgia; n (%)	984 (47.7)	636 (52.7)	348 (40.6)
Fistula in CD; n (%)		0.4 (0.48)	
Fistula surgery in CD; n (%)		321 (26.6)	
Patients with diarrhoea; n (%)	1378 (66.7)	820 (68)	558 (65)
Abdominal mass in CD; n (%)		507 (42)	
Abdominal tenderness in UC; n (%)			349 (40.7)
Nightly diarrhoea in UC; n (%)			296 (34.5)
Blood in stool in UC; n (%)			448 (52.2)
Stool incontinence in UC; n (%)			157 (18.3)
Abdominal mass in CD; n (%)		507 (42)	
Abdominal tenderness in UC; n (%)			349 (40.7)
Nightly diarrhoea in UC; n (%)			296 (34.5)
Blood in stool in UC; n (%)			448 (52.2)
Stool incontinence in UC; n (%)			157 (18.3)

Statistical analysis: *t*-test, Fisher's exact test, Chi-squared test

*n* number, *sd* standard deviation

### Selection of IBS-associated SNPs

In the SIBDC data base, data relating to 379 SNPs are available (Additional file 2). IBS genetics has been assessed in three GWAS and two meta-analysis [16–20]. Of all SNPs identified in these studies, 16 SNPs associated with IBS were also present in the SIBDC data base (Table 2).

**Table 2:** Analysed single-nucleotide polymorphisms

Name	A1A2^a^	Associated gene	Chromosome	Reference
rs1062613	CT	5-HT3A (5-hydroxytryptamine receptor 3E)	11	[20]
rs12702514	CT	KDELR2 (ER lumen protein retaining receptor 2)	7	[16]
rs1800795	GC	Il6 (Interleukin 6)	7	[19, 20]
rs324420	CA	FAAH (Fatty acid amide hydrolase)	1	[20]
rs4663866	AC	HES6 (Hes family bHLH transcription factor 6)	2	[16]
rs1042173	AC	SLC64A (Solute carrier family 6)	17	[20]
rs1042713	GA	ADRB2 (Beta-2-adrenergic receptor)	5	[20]
rs110402	GA	CRHR1 (Corticotropin-releasing hormone receptor 1)	17	[20]
rs2020936	AG	SLC64A (Solute carrier family 6)	17	[20]
rs242924	TG	CRHR1 (Corticotropin-releasing hormone receptor 1)	17	[20]
rs3779250	CT	CRHR1 (Corticotropin-releasing hormone receptor 2)	7	[20]
rs4680	GA	COMT (Catechol-O-methyltransferase)	22	[20]
rs6269	AG	COMT (Catechol-O-methyltransferase)	22	[20]
rs6311	CT	HTR2A (5-hydroxytryptamine receptor 2A)	13	[20]
rs7432532	AC	SCN5A (sodium voltage-gated channel alpha subunit 5)	3	[16]
rs2243250	CT	IL4 (Interleukin 4)	5	[16, 19, 20]

^a^A1: major allele, A2: minor allele

### Association of maximal abdominal pain with IBS-associated SNPs

**Fig. 1:**
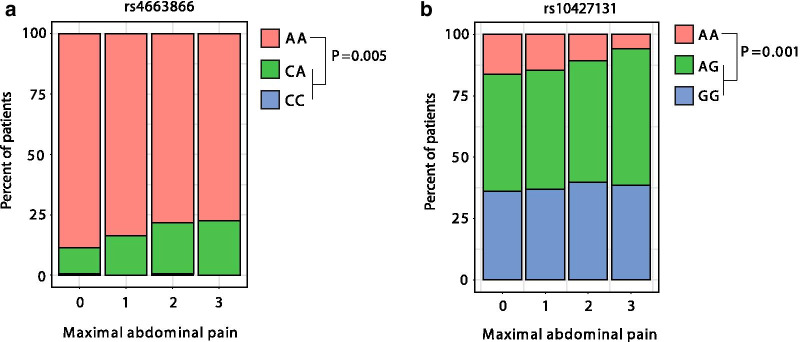
Impact of IBS-associated SNPs on abdominal pain levels in patients with UC. Pain levels according to allele status of **a** rs4663866 and **b** rs1042713. *Statistical analysis: univariate linear regression analysis*

We tested the 16 IBS-associated SNPs (Table 2) for effects on our primary endpoint maximal abdominal pain. In a univariate analysis (Additional file 1: Table S1), rs4663866 (close to the HES6 gene) was strongly associated with maximal abdominal pain in UC patients (*P* = 0.005). This association remained significant (*q* < 0.1) after the false-discovery rate was controlled by the Benjamini–Hochberg procedure (*q* < 0.09, Fig. 1a). The relationship of maximal abdominal pain with rs4663866 genotype CA/CC compared to AA remained significant in a multivariate ANOVA analysis controlling for covariates (*P* = 0.037).

Our analysis also showed a significant univariate association between rs1042713 (ADRB2 gene, allele GG and AG; *P* = 0.012, q = 0.09) and the maximal abdominal pain levels in UC patients (Fig. 1b). This association also remained significant in a multivariate ANOVA test (*P* = 0.044). Details of the difference in outcome according to the genotype (contrast in pain level) of rs4663866 and rs1042713 are shown in Table 3.

**Table 3:** Significant single-nucleotide polymorphisms

SNP	Outcome	Contrast	Slope estimate	Lower 95% CI	Upper 95% CI	*P*-value
rs4663866 (AA – CA/CC)	Maximal abdominal pain	− 0.17	0.082	− 0.33	− 0.010	0.037
rs1042713 (AA – AG—GG)	Maximal abdominal pain	− 0.18	0.088	0.35	− 0.005	0.044

Difference in outcome according to respective genotype and outcome. Reading example: our model predicts that patients with AA status at the rs4663866 loci have, on average, 0.17 lower maximal abdominal pain than those patients with CA/CC status at the rs4663866 loci. *Statistical analysis: model-based t-test (contrasts)*

In a sensitivity analysis, we restricted our analysis to SNPs for which the association with IBS was identified by a GWAS. Since rs1042713 was not identified in a GWAS, such a stringent SNP selection would limit our results to rs4663866.

In additional sensitivity analyses, we also tested the association of median abdominal pain (instead of maximal abdominal pain) and the binary variable pain level (no or mild pain versus moderate or severe pain) with the selected SNPs. This yielded in similar results for rs4663866 (median pain: *P* = 0.001, pain level: *P* = 0.011) but showed no significance for rs1042713 (median pain: *P* = 0.657, pain level: *P* = 0.089).

The univariate analysis of the selected SNPs with maximal abdominal pain in patients with CD showed nominal significance for four SNPs (rs1062613, rs3779250, rs4680 and rs6311), which was lost after applying the Benjamini–Hochberg correction (q > 0.1) (Additional file 1: Table S1).

### Effects of abdominal pain on disease management in IBD patients

**Fig. 2:**
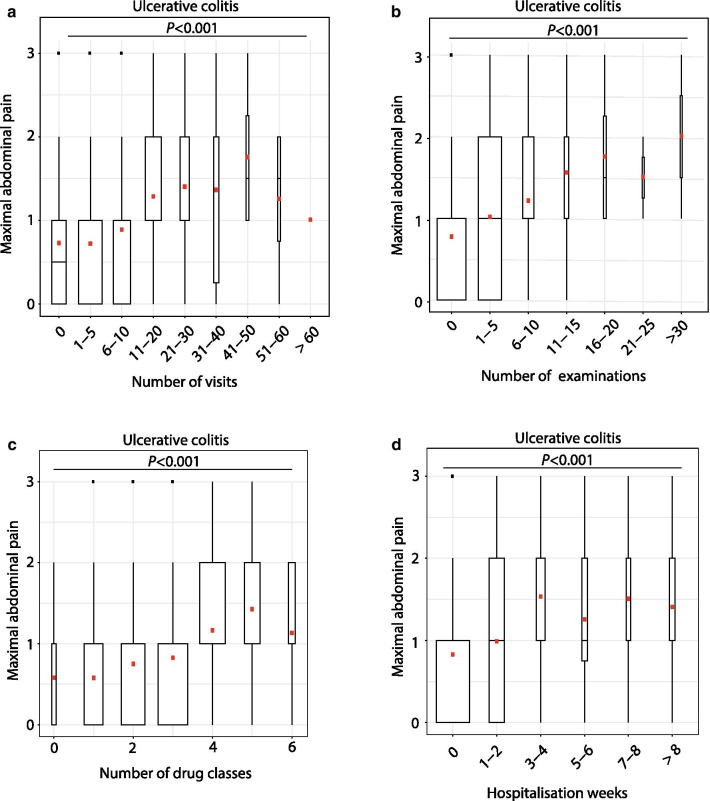
Effect of abdominal pain on disease management in patients with UC. Impact of pain on **a** number of visits, **b** number of examinations (ultrasound, endoscopy, X-ray, MRI, CT-scan), **c** cumulative number of drug classes prescribed and **d** number of weeks spent in hospital. *Statistical analysis: univariate linear regression analysis*

**Fig. 3:**
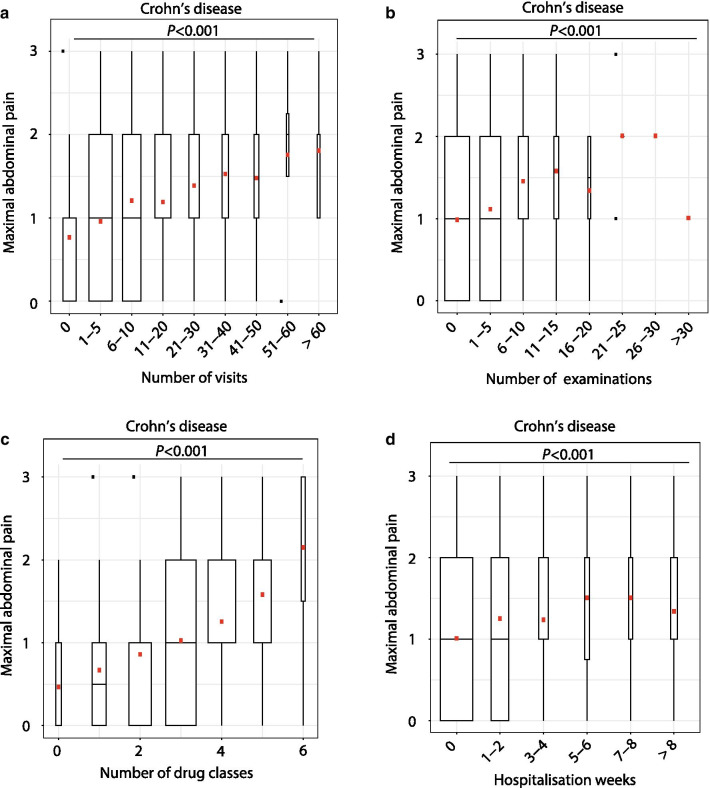
Effect of abdominal pain on disease management in patients with CD. Impact of pain on **a** number of visits, **b** number of examinations (ultrasound, endoscopy, X-ray, MRI, CT-scan), **c** cumulative number of drug classes prescribed and **d** number of weeks spent in hospital. *Statistical analysis: univariate linear regression analysis*

The degree of abdominal pain was strongly associated with the management of IBD patients in a univariate analysis. Patients with severe pain had more clinical visits, more examinations, a higher number of medical therapies and spent more weeks hospitalised (Figs. 2, 3).

**Fig. 4:**
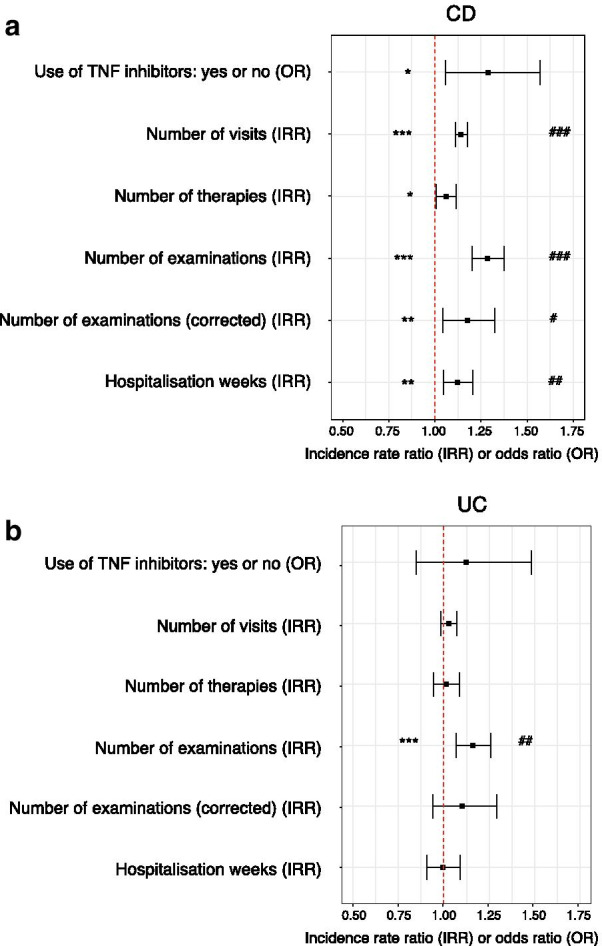
Abdominal pain levels strongly influence patient management in CD but not in UC patients. Forrest plot for the effect of abdominal pain on the indicated endpoints for **a** CD, **b** UC. Statistical analysis: multivariate logistic or Poisson regression models. Nominal associations: *P < 0.05, **P < 0.01, ***P < 0.001; Bonferroni corrected associations for confounders within the respective models: ^#^*P* < 0.05, ^##^*P* < 0.01, ^###^*P* < 0.001

In a multivariate analysis controlling for confounders, medical management remained strongly associated with pain in CD (Fig. 4a) but less in UC patients (Fig. 4b). The level of abdominal pain in CD patients was associated with the number of visits during the observation period (incidence rate ratio (IRR) 1.14, *P* < 2 × 10^–16^; Additional file 1: Table S2). However, in the UC cohort no such relationship was detectable.

Maximal abdominal pain was also an independent and highly significant predictor for the number of examinations (IRR 1.16, *P* = 4.2 × 10^–4^ for UC; IRR 1.29, *P* = 1.8 × 10^–18^ for CD; Additional file 1: Table S3). However, other clinical variables including ethnicity, depression and EIM were also associated with the number of examinations. When the number of examinations was adjusted for the number of visits, maximal abdominal pain was no longer a significant predictor in UC patients, whereas in the CD cohort the association remained robust (IRR 1.10, *P* = 0.24 for UC; IRR 1.17, *P* = 0.007 for CD).

In a simplified assessment of the number of medical therapies in IBD patients, we considered six classes of drugs (see methods). For patients with UC and CD, in a univariate analysis, individuals with a higher degree of abdominal pain had been exposed to a higher number of different treatments (*P* < 2.0 × 10^–16^ for UC, *P* < 2.0 × 10^–16^ for CD, Figs. 2c, 3c). However, in a multivariate analysis, maximal pain remained an independent predictor for the number of therapies only in CD (IRR 1.06, *P* = 0.023; Additional file 1: Table S4), but not in UC. In a sub-analysis, maximal abdominal pain showed a significant association with the use of TNF-inhibitors only in CD patients (odds ratio, OR 1.29, *P* = 0.012), but not in UC patients (data not shown).

Finally, after controlling for confounders maximal pain remained a predictor for hospitalisation weeks only in CD (IRR 1.12, *P* = 0.001; Additional file 1: Table S5), but not in UC.

### IBS-associated SNPs do not influence disease course or management of IBD patients

When IBS-associated SNPs were tested as predictors for clinical endpoints including disease activity, number of visits, examinations, hospitalisation weeks, medical therapies and TNF-inhibitor usage, no significant association robust to Bonferroni or Benjamini–Hochberg correction could be identified (data not shown).

## Discussion

We used data of 2063 patients from the Swiss IBD cohort study to analyse pain in IBD patients. The risk alleles of two IBS-associated SNPs increased maximal pain intensity experienced by UC but not CD patients. IBD patients frequently experience abdominal pain; however, in UC patients pain did not significantly influence management of IBD after controlling for confounders. In contrast, in CD patients, pain was associated with a higher number of physician contacts, examinations, medical therapies and hospitalisation weeks in a multivariate analysis. Both IBS-associated SNPs did not influence patient management.

Pain in IBD might be caused by inflammatory and non-inflammatory pathways. The genetic risk for IBD is mediated by a number of genetic regions [2–4]. However, symptoms of IBD might also be shaped by additional genetic regions associated with IBS, since some pathways of the pathogenesis of both conditions are shared [21–26]. Our analysis suggests for a role of rs4663866 and rs1042713 in mediating the level of pain experienced by IBD patients. Of note, our study does not distinguish between pain due to inflammatory and non-inflammatory conditions. Future studies need to demonstrate whether these genetic polymorphisms affect patient perception of inflammatory pain and/ or patient predisposition to develop post-inflammatory IBS (IBD-IBS).

SNP rs1042713 is located on the ADRB2 gene which encodes the beta-2-adrenergic receptor. This receptor plays an important role in pain signalling cascades [39, 40] and polymorphisms in rs1042713 affected agonist-induced receptor downregulation in vitro [41]. Rs1042713 has been associated with pain, consultations and hospitalisations in sickle cell disease [42, 43], temporomandibular pain [44], fibromyalgia [45] and chronic widespread pain [46].

The second SNP associated with abdominal pain, rs4663866, was identified in a GWAS for IBS [16]. rs4663866 was associated with the risk for IBS with nominal but not genome-wide significance (*P* = 5 × 10^–6^). SNP rs4663866 is located near the transcription factor HES6 gene but no studies addressing rs4663866 function could be found in literature and mechanistic aspects remain unclear.

We would like to emphasise that testing for post-inflammatory IBS in our study has not been possible for two reasons: First, Rome IV criteria for our patients were not available, preventing a formal diagnosis of IBS in our IBD cohort. Second, the inflammatory state (level of inflammation) has not been rigorously tested in our patients at the time of pain assessment.

In our study, abdominal pain was a crucial predictor for the intensity of disease management in CD patients. Maximal abdominal pain was strongly associated with the number of visits and investigations, TNF-inhibitor usage and overall medical therapies as well as the length of time spent hospitalised. In other studies, patient management has also been associated with measures different from acute bowel inflammation: In a longitudinal study, IBS like symptoms in IBD increased the number of clinical visits and examinations during a follow up period of more than 2 years [47]. Furthermore, a recent large cross-sectional study reported an association between IBS-like symptoms and the use of narcotics, clinical visits and the use of some medication after controlling for a limited number of confounders [48].

In our study, pain influenced clinical management in CD but only to a much lower degree in UC. A potential explanation could be the easier clinical assessment of disease activity in UC patients. The rectum is uniformly affected in UC and disease activity directly correlates with the number of bowel movements and bloody diarrhoea. The partial Mayo score (consisting of stool frequency, frequency of bloody stools and physician’s global assessment) is an accepted and validated endpoint in clinical studies of UC [49]. Of note, none of the considered IBS-SNPs were associated with IBD management. Therefore, physicians seem to rely on non-ambiguous signs of intestinal inflammation for decisions and genetic changes mediating pain levels might not significantly influence the clinical management.

In contrast, in CD, assessment of disease activity is complex and inflammation especially in the small intestine will not necessarily result in non-ambiguous symptoms, requiring usage of “softer” parameters such as the intensity of abdominal pain for treatment decisions.

Besides pain, our study also identified well-known predictors of severe disease contributing to more intense disease management. EIM were uniformly associated with all endpoints of disease management in both, UC and CD. While abdominal pain was the single symptom most consistently associated with disease management in CD, in UC, the severity of diarrhoea predicted intense management better. Other clinical markers of disease activity such as bloody diarrhoea, abdominal mass and incontinence influenced some but not all outcomes.

Our study has several strengths and limitations. To the best of our knowledge, this study is the first to analyse the association between abdominal pain, SNPs previously analysed in the context of IBS and management and disease course in IBD patients. Due to the extensive data of our cohort, we were able to test and control for many possible epidemiological, sociodemographic and clinical confounders. Limitations include lack of testing for Rome IV criteria (see above). Also, the number of IBS-related SNPs tested in the Swiss IBD cohort is limited and not all SNPs identified by GWAS and systematic reviews were available. If selection of SNPs would be limited to SNPs with an association to IBS identified by a GWAS, only rs4663866 would remain. For instance, none of the SNPs tested were associated with pain in CD and it is possible that pain in CD is mediated by other SNPs. Our study should therefore be considered as pilot study and additional analyses with more SNPs are warranted. Further, effects of rs1042713 and rs4663866 should be confirmed in an independent cohort. No validated pain questionnaires (such as the McGill Pain Questionnaire [50]) could be used because these scores had not been collected in the SIBDC. A questionnaire dedicated to the experience of pain had been used only once in the SIBDC [6] and was filled only by a subset of patients. Therefore, we preferred to use the pain items in the CDAI and the MTWAI which were repeatedly acquired. The individual perception of pain is based on complex physical mechanisms depending on various psychosocial parameters [51] and ultimately remains a highly subjective experience of an individual. Taking this into account, the pain items of the CDAI and the MTWAI might only partially reflect the level of abdominal pain experienced by patients as they are filled out by physicians, based on patient’s reports.

## Conclusions

Our study shows an association between pain levels in UC patients and IBS-associated SNPs, providing genetic evidence for an overlap of some disease mechanisms in IBD and IBS. We did not find an significant association between abdominal pain and patient management in patients with UC. However, in CD patients, pain was a strong predictor for intense patient management with regard to clinical visits, investigations and medical therapies, indicating that physicians use pain levels as a surrogate for disease activity in CD.

## Supplementary Information

**Additional file 1.** Supplementary tables.

**Additional file 2.** List of SNPs available in the SIBDC (2020).

## Data Availability

The data that support the findings of this study are available from the SIBDC but restrictions apply to the availability of these data, which were used under license for the current study, and so are not publicly available. Data are however available from the authors upon reasonable request and with permission from the SIBDC.

## Abbreviations

AIC: Akaike information criterion
ANOVA: Analysis of variance
ADRB2: Beta-2-adrenergic receptor
CDAI: Crohn's disease activity index
CNR1: Cannabinoid type 1 receptor
CT: Computer tomograpy
EIM: Extraintestinal manifestations
ER: Endoplasmic reticulum
FAAH: Fatty acid amide hydrolase
FGID: Functional gastrointestinal disorders
GWAS: Genome wide association studies
HADS: Hospital anxiety and depression scale
HES6: Hes family bHLH transcription factor 6
IBD: Inflammatory bowel disease
IBDQ: Inflammatory disease questionnaire
IBS: Irritable bowel syndrom
IL6: Interleukin 6
KDELR2: ER lumen protein retaining receptor 2
LD: Linkage disequilibrium
LS: Least square
MALDI-TOF–MS: Matrix-Assisted Laser Desorption/Ionisation–Time of Flight Mass Spectrometry
MRI: Magnetic resonance imaging
MTWAI: Modified Truelove and Witts activity index
NPSR1: Neuropeptide S receptor
QoL: Quality of life
OR: Odds ratio
SF-36: Short Form 36 Health Survey
SIBDC: Swiss IBD Cohort Study
SNF: Swiss National Science Foundation
SNPs: Single nucleotide polymorphisms
TNF: Tumor necrosis factor
TRPV1: Transient receptor potential cation channel subfamily V member 1
UC: Ulcerative colitis
