# Supplementary material for: Abdominal pain in patients with inflammatory bowel disease: association with single-nucleotide polymorphisms prevalent in irritable bowel syndrome and clinical management
Source: BMC Gastroenterol. 2021 Feb 5;21:53. doi: 10.1186/s12876-021-01622-x (PMC7866750; doi:10.1186/s12876-021-01622-x)
Supplement: Supplementary file 1 — Additional file 1. Supplementary tables. [file 12876_2021_1622_MOESM1_ESM.docx]

## Supplementary tables

| **Abdominal pain - SNPs in IBD patients** | | | | |  |
| --- | --- | --- | --- | --- | --- |
|  | CD | | UC | | |
|  | Slope estimate | P-value | Slope estimate | P-value | |
| rs1042173 CC and CA | -0.26 | 0.80 | -0.23 | 0.82 | |
| rs1042713 GG and AG | -0.04 | 0.97 | 2.53 | **0.012** # | |
| rs1062613 CT and TT | 2.80 | **0.01** | -1.66 | 0.10 | |
| rs110402 GG | 1.88 | 0.06 | 1.72 | 0.09 | |
| rs1800795 GG | 0.96 | 0.33 | 1.36 | 0.17 | |
| rs2020936 GG and AG | 0.77 | 0.44 | 0.14 | 0.89 | |
| rs2243250 CT and TT | -0.81 | 0.42 | 0.20 | 0.84 | |
| rs242924 TT | -0.51 | 0.61 | -1.57 | 0.12 | |
| rs324420 CC | -1.60 | 0.11 | 0.06 | 0.95 | |
| rs3779250 TT | -2.28 | **0.02** | -0.14 | 0.89 | |
| rs4680 GG | 2.11 | **0.03** | 0.25 | 0.81 | |
| rs6269 GG and GA | 0.54 | 0.59 | 0.34 | 0.74 | |
| rs6311 TC and TT | -2.28 | **0.02** | 0.24 | 0.81 | |
| rs12702514 CT and TT | -0.20 | 0.84 | -1.28 | 0.20 | |
| rs4663866 CC and CA | 0.07 | 0.95 | 2.80 | **0.005** # | |
| rs7432532 CA | -0.54 | 0.59 | 1.22 | 0.22 | |

**Supplementary Table S1: Predictive univariate linear model.** Bold print indicates uncorrected significance, # indicates Benjamini Hochberg–corrected p-value <0.1.

| **Number of visits** | | | | |
| --- | --- | --- | --- | --- |
|  | CD (n=720) | | UC (n=627) | |
|  | IRR | P-value | IRR | P-value |
| Maximal abdominal pain (0-3) | 1.14 | <2E-16 # | n/a | n/a |
| Gender (female) | 1.16 | 1.7E-09 # | n/a | n/a |
| Ethnicity (Caucasian) | 1.20 | 9.1E-09 # | 0.92 | 0.029 |
| Education (higher or university) | n/a | n/a | 0.89 | 0.00027 # |
| Ever smoke (yes) | n/a | n/a | 1.12 | 0.00025 # |
| Alcohol (often) | 1.26 | 3.5E-08 # | n/a | n/a |
| Depression (yes) | 1.26 | 1.7E-08 # | n/a | n/a |
| Anxiety (yes) | 0.89 | 0.00065 # | n/a | n/a |
| Extraintestinal manifestation (yes) | 1.22 | 7.0E-13 # | 1.25 | 4.4E-13 # |
| Fistula (yes) | 1.50 | <2E-16 # | n/a | n/a |
| Bloody stools (yes) | n/a | n/a | 1.13 | 1.4E-06 # |
| Stool incontinence (yes) | n/a | n/a | 1.15 | 5.22E-05 # |
| Diarrhoea (yes) | n/a | n/a | 1.16 | <2E-16 # |

**Supplementary Table S2: Predictive Poisson regression of number of visits in IBD patients.** # indicates Bonferroni-corrected p-value <0.05. IRR: Incidence rate ratio

| **Number of examinations** | | | | |  |
| --- | --- | --- | --- | --- | --- |
|  | CD (n=762) | | UC (n=596) | | |
|  | IRR | P-value | IRR | P-value |  |
| Maximal abdominal pain (0-3) | 1.29 | 1.8E-13 # | 1.16 | 0.00042 # |  |
| Gender (female) | 1.27 | 1.4E-05 # | n/a | n/a |  |
| Ethnicity (Caucasian) | 0.74 | 4.2E-07 # | 0.65 | 9.2E-10 # |  |
| Alcohol (often) | 1.60 | 6.8E-07 # | n/a | n/a |  |
| Depression (yes) | 1.29 | 0.0042 | 1.43 | 0.00079 # |  |
| Anxiety (yes) | 0.79 | 0.0011 # | n/a | n/a |  |
| Extraintestinal manifestation (yes) | 1.71 | 2.4E-16 # | 1.44 | 2.8E-08 # |  |
| Fistula (yes) | 1.51 | 3.2E-15 # | n/a | n/a |  |
| Abdominal mass (yes) | 1.18 | 0.0066 | n/a | n/a |  |
| Diarrhoea (yes) | n/a | n/a | 1.18 | 1.2E-12 # |  |

**Supplementary Table S3: Predictive Poisson regression of number of examinations in IBD patients.** # indicates Bonferroni-corrected p-value <0.05. IRR: Incidence rate ratio

| **Number of medical treatments** | | | | |
| --- | --- | --- | --- | --- |
|  | CD (n=787) | | UC (n=613) | |
|  | IRR | P-value | IRR | P-value |
| Maximal abdominal pain (0-3) | 1.06 | 0.023 | n/a | n/a |
| Extraintestinal manifestation (yes) | 1.10 | 0.033 | 1.14 | 0.0050 # |
| Abdominal mass (yes) | 1.08 | 0.075 | n/a | n/a |
| Diarrhoea (yes) | 1.01 | 0.043 | 1.24 | 2.5E-05 # |
| Bloody stools (yes) | n/a | n/a | 1.11 | 0.048 |

**Supplementary Table S4: Predictive Poisson regression of number of medical treatments used during study period in IBD patients.** # indicates Bonferroni-corrected p-value <0.05. IRR: Incidence rate ratio

| **Hospitalisation weeks** | | | | |
| --- | --- | --- | --- | --- |
|  | CD (n=709) | | UC (n=626) | |
|  | IRR | P-value | IRR | P-value |
| Maximal abdominal pain (0-3) | 1.12 | 0.0013 # | n/a | n/a |
| Depression (yes) | n/a | n/a | 1.51 | 9.3E-05 # |
| Extraintestinal manifestation (yes) | n/a | n/a | 1.12 | 0.078 |
| Fistula (yes) | 1.29 | 6.38E-06 # | n/a | n/a |
| Abdominal mass (yes) | 1.14 | 0.041 | n/a | n/a |
| Diarrhoea (yes) | 1.01 | 0.029 | 1.16 | 1.5E-07 # |

**Supplementary Table S5: Predictive Poisson regression of number of hospitalisation weeks in IBD patients.** # indicates Bonferroni-corrected p-value <0.05. IRR: Incidence rate ratio
