# Supplementary material for: Abdominal pain in patients with inflammatory bowel disease: association with single-nucleotide polymorphisms prevalent in irritable bowel syndrome and clinical management
Source: BMC Gastroenterol. 2021 Feb 5;21:53. doi: 10.1186/s12876-021-01622-x (PMC7866750; doi:10.1186/s12876-021-01622-x)
Supplement: Supplementary file 2 — Additional file 2. List of SNPs available in the SIBDC (2020). [file 12876_2021_1622_MOESM2_ESM.docx]

**Single nucleotide polymorphisms available in the SIBDC (2020)**

| rs10065637 |
| --- |
| rs1013442 |
| rs1016883 |
| rs10181042 |
| rs102275 |
| rs1028936 |
| rs1042058 |
| rs1042173 |
| rs1042713 |
| rs1042778 |
| rs10482605 |
| rs10486483 |
| rs10495903 |
| rs1051730 |
| rs10521318 |
| rs10521432 |
| rs1062613 |
| rs10733113 |
| rs10758669 |
| rs10761659 |
| rs10781499 |
| rs10797432 |
| rs10835210 |
| rs10865331 |
| rs10896794 |
| rs11010067 |
| rs11030101 |
| rs11030103 |
| rs110402 |
| rs11150589 |
| rs11167764 |
| rs11168249 |
| rs11209026 |
| rs11230563 |
| rs1126510 |
| rs113487579 |
| rs1137070 |
| rs1142287 |
| rs11564258 |
| rs11612508 |
| rs11661134 |
| rs11672983 |
| rs11676348 |
| rs11739663 |
| rs11741861 |
| rs11742570 |
| rs11871801 |
| rs11879191 |
| rs12103 |
| rs121918345 |
| rs12199775 |
| rs12242110 |
| rs12261843 |
| rs12273539 |
| rs1250546 |
| rs1250550 |
| rs12521868 |
| rs12526196 |
| rs12568930 |
| rs12584920 |
| rs12654778 |
| rs12654812 |
| rs12663356 |
| rs12702514 |
| rs12720356 |
| rs12722489 |
| rs12722515 |
| rs12914008 |
| rs1292053 |
| rs12942547 |
| rs12946510 |
| rs1297265 |
| rs12994997 |
| rs1304100 |
| rs13073817 |
| rs13140464 |
| rs13204742 |
| rs13222291 |
| rs1329650 |
| rs13428812 |
| rs1360780 |
| rs1363907 |
| rs137958968 |
| rs1379928 |
| rs139104022 |
| rs139837495 |
| rs140700 |
| rs140701 |
| rs144604969 |
| rs1456896 |
| rs1465107 |
| rs146832412 |
| rs151181 |
| rs1517352 |
| rs1569723 |
| rs165599 |
| rs16940202 |
| rs16967103 |
| rs16969968 |
| rs17085007 |
| rs17119 |
| rs17229285 |
| rs1728785 |
| rs1728918 |
| rs17289394 |
| rs17293632 |
| rs17309827 |
| rs1734907 |
| rs1736020 |
| rs17388568 |
| rs17391694 |
| rs17694108 |
| rs17695092 |
| rs1788097 |
| rs1799836 |
| rs1799971 |
| rs1800469 |
| rs1800795 |
| rs1801133 |
| rs1801274 |
| rs180955 |
| rs181359 |
| rs1819333 |
| rs1819658 |
| rs1847472 |
| rs1880676 |
| rs1893217 |
| rs194749 |
| rs1991866 |
| rs1998598 |
| rs200149900 |
| rs2020936 |
| rs2024092 |
| rs2053044 |
| rs2058660 |
| rs2062305 |
| rs2064070 |
| rs2066844 |
| rs2066845 |
| rs2066847 |
| rs2072743 |
| rs2076756 |
| rs2111485 |
| rs212388 |
| rs2155219 |
| rs222747 |
| rs2227564 |
| rs2231884 |
| rs2234237 |
| rs2234245 |
| rs2235186 |
| rs2241880 |
| rs2243250 |
| rs2266959 |
| rs2267717 |
| rs2268490 |
| rs2270007 |
| rs2283725 |
| rs2284553 |
| rs2297441 |
| rs2302009 |
| rs2310173 |
| rs237887 |
| rs2382817 |
| rs2412970 |
| rs2413583 |
| rs242924 |
| rs2472649 |
| rs2476601 |
| rs2488389 |
| rs2522411 |
| rs2522833 |
| rs254560 |
| rs2549794 |
| rs25528 |
| rs25532 |
| rs259964 |
| rs2651244 |
| rs26528 |
| rs267939 |
| rs2790216 |
| rs2797685 |
| rs281379 |
| rs2816958 |
| rs2823286 |
| rs2836878 |
| rs2836883 |
| rs28374715 |
| rs2838519 |
| rs2872507 |
| rs2930047 |
| rs2945412 |
| rs3024505 |
| rs3025343 |
| rs3091315 |
| rs3091316 |
| rs3184504 |
| rs3194051 |
| rs3197999 |
| rs324420 |
| rs33996649 |
| rs34727391 |
| rs35675666 |
| rs35753505 |
| rs359457 |
| rs3733829 |
| rs3748816 |
| rs3749171 |
| rs3764147 |
| rs3769671 |
| rs3774959 |
| rs3777747 |
| rs3779250 |
| rs3783641 |
| rs3792109 |
| rs3794808 |
| rs3800373 |
| rs3806932 |
| rs3810936 |
| rs3825427 |
| rs3851228 |
| rs38904 |
| rs3897478 |
| rs3939286 |
| rs4074134 |
| rs4077515 |
| rs4105144 |
| rs41282918 |
| rs4147359 |
| rs415890 |
| rs4246215 |
| rs4246905 |
| rs4256159 |
| rs4353135 |
| rs4380874 |
| rs4409764 |
| rs4510766 |
| rs4583306 |
| rs4606 |
| rs4633 |
| rs4656940 |
| rs4663866 |
| rs4676406 |
| rs4680 |
| rs4713902 |
| rs4713916 |
| rs4722672 |
| rs4728142 |
| rs4743820 |
| rs4802307 |
| rs4809330 |
| rs4818 |
| rs4836519 |
| rs483905 |
| rs4845604 |
| rs4871611 |
| rs4899554 |
| rs4902642 |
| rs4911259 |
| rs4923457 |
| rs4923460 |
| rs516246 |
| rs529866 |
| rs53576 |
| rs55646866 |
| rs559928 |
| rs561722 |
| rs56258221 |
| rs5771069 |
| rs578776 |
| rs588765 |
| rs5906957 |
| rs5953210 |
| rs6017342 |
| rs6062504 |
| rs60652743 |
| rs6088765 |
| rs6142618 |
| rs61816761 |
| rs618167611 |
| rs61888800 |
| rs6198 |
| rs6265 |
| rs6269 |
| rs630923 |
| rs6311 |
| rs6323 |
| rs6354 |
| rs6426833 |
| rs6451493 |
| rs6474412 |
| rs6484320 |
| rs6499188 |
| rs6545800 |
| rs6556412 |
| rs6568421 |
| rs6584283 |
| rs6586030 |
| rs6592362 |
| rs6651252 |
| rs6651806 |
| rs6679677 |
| rs670523 |
| rs6716753 |
| rs6738825 |
| rs6740462 |
| rs6746030 |
| rs6771148 |
| rs678170 |
| rs6837335 |
| rs6863411 |
| rs6871626 |
| rs6908425 |
| rs6911490 |
| rs6918698 |
| rs6920220 |
| rs6927022 |
| rs694739 |
| rs7015630 |
| rs7016778 |
| rs7134599 |
| rs7210086 |
| rs7240004 |
| rs727088 |
| rs7282490 |
| rs734999 |
| rs736289 |
| rs7404095 |
| rs740495 |
| rs7423615 |
| rs7426056 |
| rs7432532 |
| rs7495132 |
| rs7517810 |
| rs7554511 |
| rs7608910 |
| rs7657746 |
| rs7702331 |
| rs7714584 |
| rs780093 |
| rs7824175 |
| rs7911264 |
| rs7927997 |
| rs7937682 |
| rs798502 |
| rs7999348 |
| rs8005161 |
| rs8007267 |
| rs8022616 |
| rs806378 |
| rs8076005 |
| rs8192466 |
| rs846910 |
| rs852977 |
| rs864745 |
| rs879048 |
| rs907611 |
| rs913678 |
| rs917997 |
| rs921720 |
| rs925255 |
| rs9264942 |
| rs9286879 |
| rs9297145 |
| rs9358372 |
| rs9399005 |
| rs941601 |
| rs941823 |
| rs943072 |
| rs9470080 |
| rs9491697 |
| rs950880 |
| rs9513584 |
| rs9517668 |
| rs9517701 |
| rs9534511 |
| rs9557195 |
| rs9822268 |
| rs9847710 |
